# 

**Published:** 1888-04

**Authors:** 


The Chicago Medical Journal and Examiner.

Volume 56. Number 4.

### CONTENTS OF THIS NUMBER.

**Original Communications—** PAGE.

Erythrophlæine as a Local Anæsthetic. Carl Koller ... 201
Nephritis Secondary to Aortic Regurgitation. J. A. Robison ... 203
Cyst of the Pancreas. D. A. K. Steele ... 205
Three Cases of Nerve-Injury—Operation—Recovery. H. H. Frothingham ... 209
Eczema Simplex Dependent upon Ametropia. S. O. Richey ... 211

**Editorial—**

The Amorphous Urate Deposit ... 212
Adulteration of Food and Medicine ... 213
The Index Medicus ... 213
Sanitary Matters in Michigan ... 214

**Society Reports—**

Transactions of the Gynæcological Society of Chicago, Regular Meeting, December 16, 1887 ... 214
A New Uterine Supporter ... 214
Tumor of the Ileum ... 214
Vaginal Hysterectomy for Sarcoma Uteri ... 216
Chicago Pathological Society, Stated Meeting, March 12 ... 224

**Foreign Medical Matters** ... 231

**Extracts and Abstracts—**

Aniline Poisoning ... 232
Japanese "Kakke" ... 238
Heathen Ignorance of the Healing Art ... 242
Inversion in Suspended Animation during Anæsthesia ... 245
Diverting the Ureters and Removing the Bladder ... 247
Operation for Abscess of the Spleen ... 249
A Case of Cholecystotomy, with Recovery ... 251
Health Rules in time of Cholera ... 253
Antiseptic and Antipiretic Treatment of Phthisis ... 254
Sulphate of Spartein ... 255
Circumcision in the Adult ... 256
Electrical Treatment in Salpingitis ... 257
Epidemiology ... 257
Pilocarpine in Bright's Disease ... 258
Subcutaneous Separation of Trachea and Larynx ... 258
Simple Puncture in Empyema ... 258
Antiseptic Pocket-Case ... 259
Inhalation of Carbonic Acid Gas in Dyspnœa ... 259

**Book Reviews** ... 259

**Books Received** ... 262

**Miscellaneous—**

Chicago's New Garbage Crematory ... 262
Annual Meeting of The Alumni of Internes of Cook County Hospital ... 262
College of Physicians and Surgeons ... 263
Chicago Medical College ... 263
Training-Schools for Attendants in Asylums for the Insane ... 263

**Announcements—**

Congress of American Physicians and Surgeons ... 264
Gari Prize on Blennorrhagia ... 264

